# Comparative Evaluation of the Montreal Cognitive Assessment Basic Scale Against the Mini-Mental State Examination for Post-Stroke Cognitive Impairment

**DOI:** 10.31083/AP39895

**Published:** 2025-04-22

**Authors:** Qingjun Zhu, Meirong Chen, Xiang Li, Lin Huang, Jinling Qiao, Miaocun Chen, Huizhi Ma

**Affiliations:** ^1^Department of Psychology & Fudan Development Institute, Fudan University, 200433 Shanghai, China; ^2^Department of Neurology, Jiangwan Hospital of Hongkou District, 200081 Shanghai, China; ^3^Department of Integrative Biology and Physiology, University of California, Los Angeles, CA 90024, USA; ^4^Department of Gerontology, Shanghai Sixth People’s Hospital Affiliated to Shanghai Jiao Tong University School of Medicine, 200030 Shanghai, China; ^5^Department of Rehabilitation, Putuo People’s Hospital, Tongji University, 200060 Shanghai, China

**Keywords:** MoCA-B, MMSE, stroke, cognitive impairment, Vascular Dementia, Alzheimer’s disease (AD)

## Abstract

**Background:**

The Montreal Cognitive Assessment Basic scale (MoCA-B) is more sensitive than the Mini-Mental State Examination (MMSE) for detecting mild cognitive impairment due to Alzheimer’s disease (AD). To explore the diagnostic efficacy of the Chinese version of the MoCA-B against the MMSE for post-stroke cognitive impairment (PSCI).

**Methods:**

Eighty four patients with acute cerebral infarction were grouped into a post-stroke cognitive normal (PSCN) or a PSCI group based on their scores on the Clinical Dementia Rating scale (CDR), the gold standard for diagnosing PSCI. They were evaluated by using the MMSE and MoCA-B scales, then the area under the receiver operating characteristic (ROC) curve (AUC) was used for evaluation.

**Results:**

Most factors of the MoCA-B were significantly different between the two groups, and the PSCN group completed the MoCA-B faster (*p* < 0.05). The AUC analysis showed that for the MoCA-B with a cut-off total score of 23, sensitivity = 85.71%, specificity = 61.22%, Youden’s J Index = 0.469, and AUC = 0.832. For the MMSE with a cut-off total score of 25, sensitivity = 70.59%, specificity = 93.75%, Youden’s J Index = 0.643, and AUC = 0.885. The AUC of the MMSE was higher than that of the MoCA-B (*p* > 0.05). The MoCA-B had greater sensitivity and negative predictive value than the MMSE. When considering the cutoffs for identifying mild cognitive impairment (MCI) across different education levels, the MoCA-B had a higher positive rate for PSCI identification (51.2% vs 25%, *p* < 0.001), indicating that the MoCA-B is suitable for identifying PSCI.

**Conclusion:**

The MoCA-B demonstrates higher sensitivity and negative predictive value compared with the MMSE in the screening of post-stroke cognitive impairment patients.

## Main Points

1. **Question**: Is the Montreal Cognitive Assessment Basic scale more effective than the MMSE in screening for cognitive impairment after stroke?

2. **Findings**: The MoCA-B showed higher sensitivity and negative predictive value than the MMSE.

3. **Significance**: Our results underscore the feasibility and superiority of MoCA-B over MMSE in screening for post-stroke cognitive impairment, thereby enhancing the clinical management of patients afflicted with this condition.

4. **Future Extensions**: Predictive models must be devised, and longitudinal studies can authenticate the significance of the MoCA-B in identifying post-stroke cognitive decline and anticipate the progression of Vascular Dementia (VaD) and/or AD occurring 3–6 months, or longer, subsequent to a stroke.

## 1. Introduction

Post-stroke cognitive impairment (PSCI) is described as a cognitive deficit occurring after a clinical stroke, without any previous major cognitive decline before the stroke. It manifests as a disorder of thinking skills, memory, visuospatial ability, language, and attention, etc. [1]. PSCI is a subset of Vascular Cognitive Impairment (VCI) and accounts for a significant proportion (20%–40%) of all dementia diagnoses [2]. The incidence of cognitive impairment in the first 6 months following a stroke event has been shown to be as high as 52% [3], with 7.4% to 41.3% of patients developing dementia [4]. Dementia, a common disease affecting 55 million people worldwide, usually occurs after PSCI. Categories of dementia include Alzheimer’s disease (AD), Vascular Dementia (VaD), and other degenerative dementias, etc. [5, 6].

The Mini-Mental State Examination (MMSE), Montreal Cognitive Assessment scale (MoCA), and Montreal Cognitive Assessment Basic scale (MoCA-B) are the main cognitive functional screening tools for evaluating dementia. The MMSE has been one of the most popular cognitive screening tools for more than 30 years [7]. The scale encompasses six cognitive domains: orientation, memory, attention, calculation, language, and visuospatial skills. A previous study found that the MMSE is a sensitive tool for detecting patients with cognitive impairment, especially those with dementia [8]. However, the MMSE exhibits several areas of potential enhancement, notably its reduced accuracy in identifying individuals with mild cognitive impairment (MCI) and its reduced sensitivity towards patients with mild AD [9]. In addition, the MMSE may present false positive outcomes for people with low education levels [10].

The MoCA is an evaluation tool for the detection of AD and MCI derived from a memory clinic population, and it is superior to the MMSE in the identification of MCI, but the cut-off of <26/30 may not be appropriate in a population with cerebrovascular disease [11]. The MoCA-B is a shorter and more simplified version of the original MoCA test. The MoCA-B is designed to be administered quickly and easily, making it suitable for use in settings where time or resources are limited. It evaluates basic cognitive functions such as memory, orientation, executive function, and other domains: attention, calculation, language, and visuospatial skill. The MoCA-B is extremely sensitive and specific for patients with MCI due to AD, cerebrovascular disease, and Parkinson’s disease, etc. [12, 13]. The MoCA-B was introduced to detect people’s cognitive function with lower educational levels. In China, the MoCA-B was proven as an effective tool for identifying Chinese elders with normal cognition, MCI, and mild to moderate AD [14, 15].

To summarize, while the focus of the MMSE lies in conventional cognitive domains, the MoCA-B evaluates a broader range of cognitive functions, potentially making it more sensitive to mild cognitive changes and AD [16, 17]. Nevertheless, there is still a lack of information regarding whether the MoCA-B performs better than the MMSE for detecting PSCI. To improve the efficiency in clinical assessment, it is worth considering if the MoCA-B could somehow replace the MMSE in the future for screening cognitive impairment after stroke, thereby streamlining the identification in clinical diagnosis. Hence, the present study’s primary aim is to test whether the MoCA-B has higher sensitivity than the MMSE for detecting cognitive impairment in a post-stroke population.

## 2. Materials and Method

### 2.1 Subjects

A total of 84 patients with acute cerebral infarction in the Department of Neurology of Putuo District People’s Hospital in 2019 were assessed using the Chinese versions of the MMSE, MoCA-B, and Clinical Dementia Rating scale (CDR) to evaluate overall cognitive function. All subjects were approved by themselves or their guardians. The study was conducted in accordance with the Declaration of Helsinki and the protocol was approved by the Ethics Committee of Shanghai Sixth People’s Hospital (approval number: 2019-041). Written informed consent was obtained from all participants or their legal representatives.

### 2.2 Inclusion Criteria

All subjects were diagnosed with acute cerebral infarction using the International Classification of Disease-10th Revision (ICD-10) for the first time. They had a complete medical history consisting of present and past illnesses; physical examination; evaluations of anxiety, depression, and other mental disorders; and neuroimaging (computed tomography (CT) or magnetic resonance imaging (MRI)). In addition, most of them underwent laboratory tests for hematological vitamin B12, folic acid (FA), and thyroid function, together with a rapid plasma reagin (RPR) circle card test and Treponema pallidum particle assay. Patients between 45 and 85 years old that were willing to finish the tests were recruited. The assessment was performed before discharge when the stroke had been stable for more than 1 week.

The cutoff of the two scales is different based on the various levels of education the subjects obtained. An MoCA-B score of ≤22 was used for patients with an education level of middle or high school, and a score of ≤24 was used for those with a university degree [18]. In this study, the diagnostic gold standard for PSCI was defined as a CDR score of >0.5 [19].

### 2.3 Exclusion Criteria

**Fig. 1.**
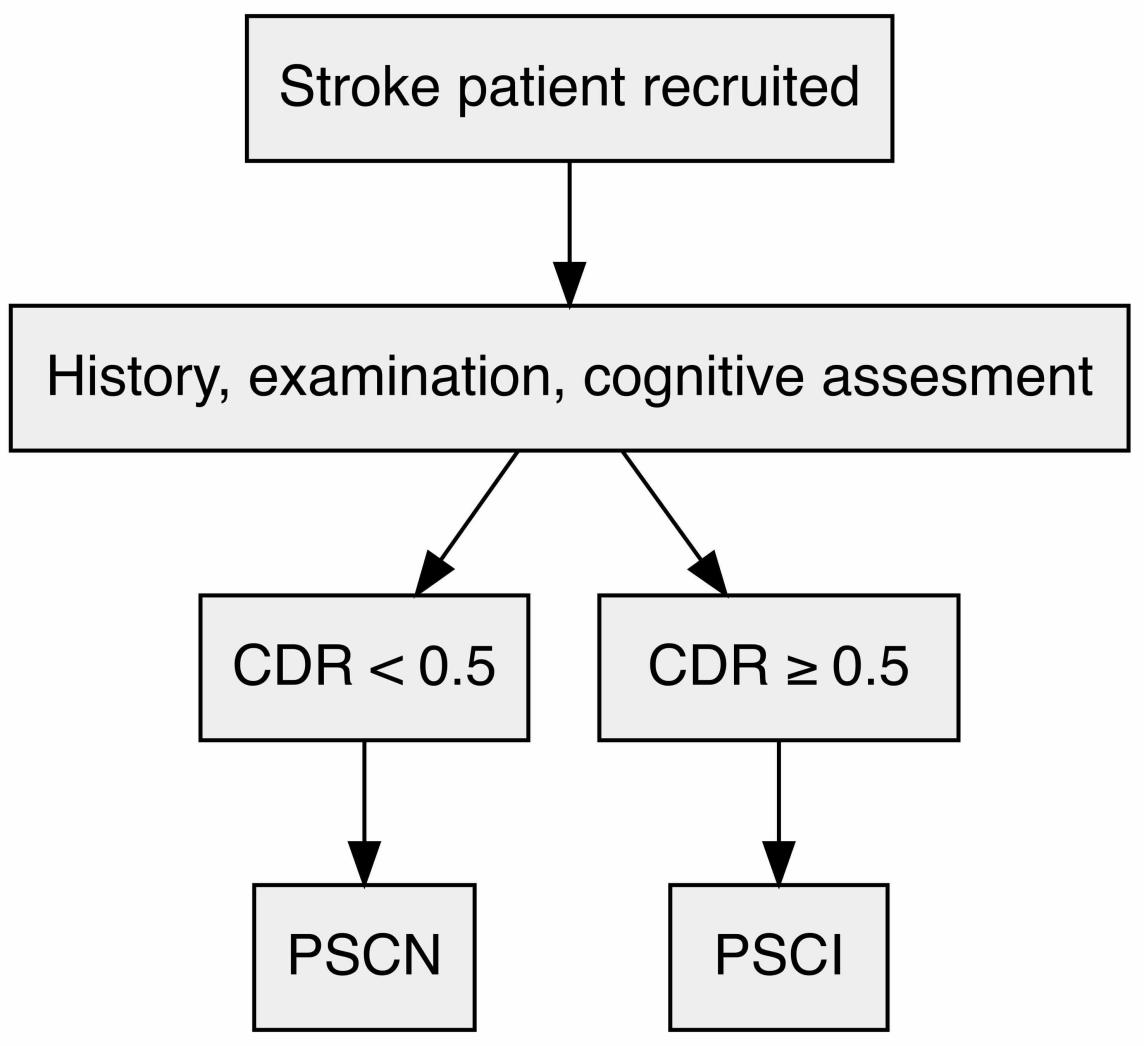
**Flow chart outlining the cognitive function screening process in post-stroke patients**. CDR, Clinical Dementia Rating scale score; PSCI, post-stroke cognitive impairment; PSCN, post-stroke cognitive normal.

Patients with any of the following were excluded from this study: severe malnutrition; infection; drug or alcohol addiction; schizophrenia; schizoaffective disorder or primary affective disorder; severe auditory, visual, or motor deficits; heart, liver, kidney, and hematopoietic system diseases; or other primary severe diseases that may interfere with cognitive testing, as shown in Fig. 1.

### 2.4 Statistical Analyses

Statistical analyses were performed using SPSS 22.0 software (v.22.0 for Windows, SPSS, IBM, Armonk, NY, USA). Gender difference comparisons between the two groups were analyzed using the chi-squared test, followed by the Shapiro-Wilk test, which indicated that the data were not normally distributed. The age and education years of the two groups were examined using the Mann-Whitney U test. The total scores for MMSE and MoCA-B, along with the factor scores of both groups, recognized as non-normally distributed, are shown in the tables, with continuous data expressed as medians (Q1–Q3) and tested using the Mann-Whitney U test.

Additionally, specificity, sensitivity, positive predictive value, and negative predictive value were evaluated using the fourfold table method. Receiver operating characteristic (ROC) curve analysis was performed to determine the optimum threshold value, Youden’s J Index, sensitivity, and specificity. The area under the ROC curve (AUC) was computed to evaluate the diagnostic performance of the MoCA-B and MMSE in screening for PSCI. Cohen’s kappa statistic was calculated to assess the level of agreement between the MoCA-B and MMSE in diagnosing PSCI. Statistical comparisons of the AUC values were performed using the Z test to select the best diagnostic parameters. The level of significance was set at *p* < 0.05.

## 3. Results

### 3.1 Demographic Characteristics

The demographic characteristics are shown in Table 1. Based on a comprehensive clinical assessment, 35 of the 84 subjects were categorized as PSCI group and the remaining 49 were post-stroke cognitively normal (PSCN group). Gender was shown as counts and percentages, while age and years of education were described using medians and interquartile ranges. There was no significant difference in gender, age, sex, and years of education between the cognitively normal group and the cognitively impaired group (*p* > 0.05).

**Table 1.** **Comparison of the demographic characteristics of the PSCI group and the PSCN group**.

		Total (n = 84)	PSCN (n = 49)	PSCI (n = 35)	*p*-value
Gender, [n (%)]					0.443^a^
	Male	49	36 (73.47%)	23 (65.71%)	
	Female	35	13 (26.53%)	12 (34.29%)	
Age, [years]					0.284^b^
	Median (Q1–Q3)	68.5 (64–72)	68 (64–71)	69 (64–77)	
Education, [years]					0.451^b^
	Median (Q1–Q3)	10.5 (9–12)	10 (9–12.5)	11 (9–12)	

^a^Chi-Squared test. ^b^Mann-Whitney U test.

### 3.2 Comparison of Cognitive Characteristics

The Shapiro-Wilk test was used to evaluate the normality of the MoCA-B and MMSE scores in the PSCN and PSCI groups, and indicated non-normal distribution. The scores were described by using medians and interquartile ranges. We then used the Mann-Whitney U test to compare the scores between the PSCN and PSCI groups. As shown in Table 2, the test revealed a significant difference in MMSE scores between the PSCN and PSCI groups (*p* < 0.001). Similarly, a significant difference was observed in the MoCA-B scores between the two groups (*p* < 0.001), showing the effectiveness of both the MMSE and MoCA-B in differentiating between cognitively normal and cognitively impaired individuals and validating their utility as diagnostic tools for assessing cognitive function.

**Table 2.** **Comparison of cognitive characteristics between the PSCN group and the PSCI group**.

	PSCN (n = 49)	PSCI (n = 35)	*p*-value
**MMSE**	Median (Q1–Q3)	Median (Q1–Q3)	
Total Score	28 (27–29)	24 (20–26)	<0.001
Instant Recall	3 (3–3)	3 (3–3)	0.097
Delayed Recall	2 (2–3)	2 (1–2)	0.001
Orientation	10 (10–10)	9 (8–9)	<0.001
Calculation/Attention	5 (4–5)	3 (1–4)	<0.001
**MoCA-B**	Median (Q1–Q3)	Median (Q1–Q3)	
Total Score	25 (22–27)	17 (15–22)	<0.001
Trail Making	0 (0–1)	0 (0–0)	0.090
Instant Recall 1	4 (3–4)	3 (2–4)	0.004
Instant Recall 2	5 (4–5)	4 (4–5)	0.002
Verbal Fluency	1 (1–2)	1 (0–1)	0.003
Verbal Fluency N	11 (9–13)	8 (6–10)	0.001
Calculation	3 (3–3)	2 (0–3)	<0.001
Delayed recall	2 (1–4)	0 (0–1)	<0.001
Orientation	6 (6–6)	6 (5–6)	0.001
Abstraction	2 (2–3)	2 (1–3)	0.062
Visuoperception	3 (2–3)	2 (1–2)	<0.001
Visuoperception N	9 (7–9)	6 (4–8)	<0.001
Animal Naming	4 (4–4)	4 (3–4)	0.004
Attention	3 (3–3)	3 (0–3)	<0.001
Time taken (s)	695 (610–875)	895 (780–1062.5)	<0.001

Note: all scores are described as medians and interquartile ranges. The *p*-value was obtained by Mann-Whitney U test. MMSE, Mini-Mental State Examination; MoCA-B, Montreal Cognitive Assessment Basic scale.

For specific tests within the MMSE and MoCA-B, the Mann-Whitney U test was also applied. The instant recall score of the MMSE was not significantly different (*p* = 0.097), while the MoCA-B scores showed differences between the groups. Most factor scores varied between groups, except for instant recall, trail making, and abstraction. The cognitively impaired group took significantly more time to complete the tests (*p* < 0.001).

### 3.3 Diagnostic Efficiency

The accuracy, sensitivity, specificity, and positive and negative predictive values are shown in Table 3. The MoCA-B had high sensitivity and negative predictive value, while the MMSE had high specificity, negative predictive value, and accuracy.

**Table 3.** **The four-grid distribution of cognitive impairment diagnosed by the MMSE and MoCA-B screening, respectively**.

	Accuracy	Sensitivity	Specificity	Positive PV	Negative PV
MMSE	80.95%	57.14%	97.96%	95.24%	76.19%
MoCA-B	73.81%	80%	69.39%	65.12%	82.93%

Positive PV, positive predictive value; Negative PV, negative predictive value.

The results of the ROC curve analysis are summarized in Table 4. The sensitivity, specificity, Youden’s J Index, and AUC were as follows: sensitivity = 70.59%, specificity = 93.75%, Youden’s J Index = 0.643, and AUC = 0.885 for a cut-off MMSE total score of <25; sensitivity = 85.71%, specificity = 61.22%, Youden’s J Index = 0.469, and AUC = 0.832 for a cut-off MoCA-B total score of <23. The AUC of the MMSE total score was greater than that of the MoCA-B total score, and the difference in the AUC between the two was not statistically significant (Z =1.152, *p* = 0.250).

**Table 4.** **ROC analysis of the MMSE and the MoCA-B for detecting cognitive impairment**.

	AUC (SE)	*p*	95% CI	Youden’s J Index	Cutoff Score	Sensitivity	Specificity
MMSE	0.885 (0.038)	<0.001	0.812–0.958	0.643	<25	70.59%	93.75%
MoCA-B	0.832 (0.044)	<0.001	0.746–0.919	0.469	<23	85.71%	61.22%

ROC, receiver operating characteristic curve; CI, confidence interval; AUC, area under the curve.

Referring to the cutoff that identifies MCI in different education levels, if the cutoff scores of the MoCA-B for PSCI screening were 19 for individuals with no more than 6 years of education, 22 for individuals with 7 to 12 years of education, and 24 for individuals with more than 12 years of education, 43 of the 84 cases were defined as PSCI. Likewise, 21 of 84 were defined as PSCI according to the cutoff scores of the MMSE, which were 17 for individuals with no more than 6 years of education, 22 for individuals with 7 to 12 years of education, and 24 for individuals with more than 12 years of education. The positive rate of 51.2% was higher than the 25% positive rate, and the difference was statistically significant (*p* < 0.001), as shown in Table 5 (chi-squared test). The kappa value, which shows the agreement between the MoCA-B and MMSE in diagnosing PSCI, was 0.341 with *p* < 0.001.

**Table 5.** **Analysis for the cognitive impairment positive rate defined by the MMSE and MoCA-B**.

Group	PSCN by MoCA-B n (%)	PSCI by MoCA-B n (%)	Total
PSCN by MMSE n (%)	38 (45.2%)	25 (29.8%)	63 (75%)
PSCI by MMSE n (%)	3 (3.6%)	18 (21.4%)	21 (25%)*
Total	41 (48.8%)	43 (51.2%)*	84

*p < 0.001.

## 4. Discussion

In the context of acute stroke, it is debatable which gold standard is the appropriate comparator [20]. This study used CDR combined with clinical data as the gold standard for clinical diagnosis. The results showed that the sensitivity of the MoCA-B is considerably higher than that of the MMSE, which indicates that the MoCA-B has higher sensitivity than the MMSE for detecting PSCI, as it does in screening for MCI [14]. Moreover, the accuracy and negative predictive value of the MoCA-B are similar to that of the MMSE and there is no statistical difference between them. Hence, the MoCA-B is suitable for clinical screening of PSCI. Regarding specificity and the positive predictive value, MoCA-B is lower than the MMSE, which is consistent with a previous study [21]. Therefore, if a patient is not suspected to have PSCI through screening, the MMSE can be used as a supplement to improve specificity. The thorough analysis, incorporating a fourfold table, revealed statistically significant variations in the outcomes of both tests, thereby underscoring the enhanced value of the MoCA-B in diagnosing PSCI.

In addition, indicators of instant recall, delayed recall, verbal fluency, orientation, animal naming, vision, and time consumption in the MoCA-B all show statistical differences between the cognitively impaired and cognitively normal groups. These indicators help in screening for PSCI, compared with the MMSE. The MoCA-B allows for more detailed observation and diagnosis of cognitive impairment. Thus, it indicated that the MoCA-B has some advantages over the MMSE in observing many indicators between the two groups. The MMSE cannot identify impaired cognitive domains except orientation, although it consists of several detailed indicators. A comparison of the two scales shows that the Instant Recall scores of the PSCI group and the PSCN group were not significantly different in the MMSE but were in the MoCA-B. A possible explanation for this result is that the Instant Recall item in the MoCA-B includes three more points than in the MMSE. As shown in the statistical results, the *p*-values of delay memory factors, both in the MMSE and MoCA-B, were much lower than 0.05, which suggests that Delayed Recall impairment occurs in people with PSCI. Indeed, subjective memory complaints (SMCs) among stroke patients are common [22]. The delayed memories factor of MoCA-B as an additional screening tool can be used.

Interestingly, there was no significant difference between the scores of Trail Making and Abstraction in the MoCA-B for the PSCI group and the PSCN group. The result was not in accordance with the characteristics of vascular cognitive impairment, which usually presents primarily as executive function impairment. In contrast, the Trail Making test is an excellent neuropsychological test for examining executive function [23]. The Trail Making item within the MoCA-B assessment might be deemed too concise, with scores limited to either 0 or 1 point, in contrast to the original Trail Making Test (TMT) version which documents the elapsed time for task completion. This limits the detection ability of TMT in the MoCA-B for identifying the cognitive impairment as an independent indicator. The original version of the TMT, using the time taken as the main scoring point, is a stable indicator to detect cognitive impairment. Also, many people have attention deficits after stroke. As attention is a multifaceted process, the assessment of attention requires the use of specific neuropsychological tests such as cross-out, symbol search, (cued) flanker, co/no-go, tone discrimination tasks, and even more complex paradigms using two tasks simultaneously (i.e., dual tasks). The time taken of MoCA-B in the PSCI group was significantly higher than in the other group, indicating that the executive function of the PSCI group was indeed worse than that of the normal group.

As it is debatable which gold standard is the most appropriate in acute stroke, and the assessment score is related to people’s education level, analysis for the cognitive impairment positive rate defined by the MMSE and MoCA-B according to education, which refers to the cutoff that identifies MCI, also showed that the MoCA-B is more sensitive. The frequency of 51.2% was similar to 47.3%, which was investigated in a study conducted 3 months post-stroke in France [24]. Therefore, the prevalence of PSCI differed between regions and races [25, 26].

Lastly, this study has some limitations. The sample size needs to be bigger, and no follow-up neuropsychological assessment or pathological analyses were carried out. Therefore, a considerably more improved setting should be used in our further studies to fully validate the usefulness and accuracy of our early screening form. Additionally, by incorporating the Neuropsychological Assessment Battery (NAB) for detailed evaluation, we could determine whether certain items in the MoCA-B might serve as substitutes for the NAB in detecting impaired cognitive domain. This approach would also enable us to develop a predictive model to identify which patients are likely to progress to dementia, thereby providing a more precise basis for cognitive rehabilitation [27].

## 5. Conclusion

In conclusion, a pathological baseline score on the MoCA-B (<23) did not predict an increased risk of cognitive decline at follow-up. The MoCA-B demonstrates feasibility and superior sensitivity compared to the MMSE in screening for post-stroke cognitive impairment, particularly when education-adjusted cutoffs are applied. If the cutoff is set according to patients’ education, the sensitivity will be increased. As a next step, longitudinal studies are required to predict and validate the value of the MoCA-B in screening the post-stroke period for cognitive impairment and the development of VaD and/or AD by formal neuropsychological evaluation 3–6 months, or longer, after stroke.

## Availability of Data and Materials

The datasets used and analyzed during the current study are available from the corresponding author upon reasonable request.
